# Variation in WNT7A is unlikely to be a cause of familial Congenital Talipes Equinovarus

**DOI:** 10.1186/1471-2350-9-50

**Published:** 2008-06-06

**Authors:** Guoqing Liu, Julie Inglis, Amanda Cardy, Duncan Shaw, Sukhy Sahota, Raoul Hennekam, Linda Sharp, Zosia Miedzybrodzka

**Affiliations:** 1 Department of Medicine and Therapeutics, University of Aberdeen, Aberdeen, UK; 2 Department of Public Health, University of Aberdeen, Aberdeen, UK; 3 School of Medical Sciences, University of Aberdeen, Aberdeen, UK; 4 Department of Paediatrics, Amsterdam Medical Centre, Amsterdam, The Netherlands; 5 Clinical and Molecular Genetics Unit, Institute of Child Health, University of London, London, UK; 6 National Cancer Registry Ireland, Cork, Ireland

## Abstract

**Background:**

Genetic factors make an important contribution to the aetiology of congenital talipes equinovarus (CTEV), the most common developmental disorder of the lower limb. WNT7A was suggested as a candidate gene for CTEV on the basis of a genome-wide scan for linkage in a large multi-case family. WNT7A is a plausible candidate gene for CTEV as it provides a signal for pattern formation during limb development, and mutation in WNT7A has been reported in a number of limb malformation syndromes.

**Methods:**

We investigated the role of WNT7A using a family-based linkage approach in our large series of European multi-case CTEV families. Three microsatellite markers were used, of which one (D3S2385) is intragenic, and the other two (D3S2403, D3S1252) are 700 kb 5' to the start and 20 kb from the 3' end of the gene, respectively. Ninety-one CTEV families, comprising 476 individuals of whom 211 were affected, were genotyped. LOD scores using recessive and incomplete-dominant inheritance models, and non-parametric linkage scores, excluded linkage.

**Results:**

No significant evidence for linkage was observed using either parametric or non-parametric models. LOD scores for the parametric models remained strongly negative in the regions between the markers, and in the 0.5 cM intervals outside the marker map. No significant lod scores were obtained when the data were analysed allowing for heterogeneity.

**Conclusion:**

Our evidence suggests that the WNT7A gene is unlikely to be a major contributor to the aetiology of familial CTEV.

## Background

Congenital talipes equinovarus (CTEV), colloquially known as "clubfoot", is a common developmental disorder of the lower limb, with an incidence of 1 – 7 per 1000 births in various populations [1]. In the UK 1–2 births per 1000 are affected [2]. CTEV is a three dimensional malformation immediately recognisable at birth; the ankle is in the plantar flexed (equinus) position, the heel is inverted (varus) and the midfoot and forefoot are inverted and adducted (varus). Epidemiological studies implicate multifactorial inheritance. Pedigree analyses have suggested a major role of a single gene, with both variably penetrant autosomal dominant and recessive patterns fitting the data [3-5]. In a systematic review of the literature we found that a family history of CTEV was present in 24–50% of cases depending on the population studied [1]. Identification and characterization of the causative gene(s) will contribute to our understanding and treatment of clubfoot as well as the determinants of normal limb growth and development.

Wnt genes encode a family of highly conserved cysteine rich glycoproteins that play an important role in the normal developmental processes during embryogenesis [6,7] and in carcinogenesis [8]. The Wnt family has at least 19 members; several of them are expressed in the limb, where they control patterning, outgrowth and/or differentiation [9]. WNT7A is known to be involved in limb development [10-13]. In mouse [10] and chicken [14], Wnt-7a provides a signal for pattern formation during limb development. In human, mutations in WNT7A cause a range of limb malformations including Fuhrmann syndrome and Al-Awadi/Raas-Rothschild/Schinzel Phocomelia syndrome, indicating the specific and conserved importance of WNT7A in multiple aspects of vertebrate limb development [13]. WNT7A is thus an excellent candidate gene for CTEV. In 2005, Dietz and co-workers reported a genome wide scan for linkage in a four generation CTEV family, comprising 13 individuals with clubfoot and 41 unaffected members [15]. The highest LOD score of 2.18 was obtained for markers on chromosome 3, close to the WNT7A gene. We therefore performed a linkage study of the WNT7A locus in our large series of European multiplex CTEV families.

## Methods

### Recruitment of families

**Figure 1:**
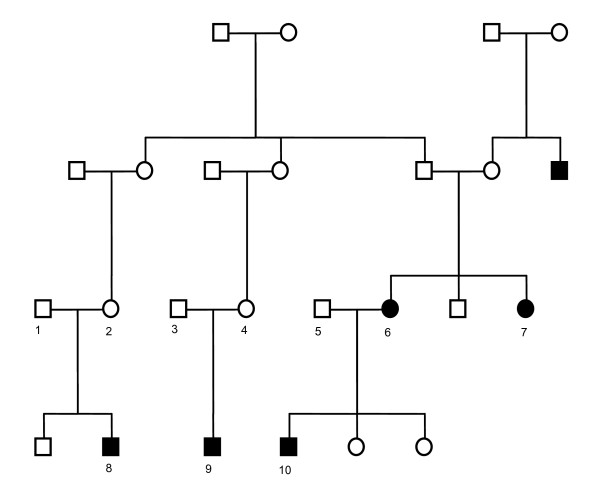
**Pedigree of an informative Dutch CTEV family used in this study**. Individuals affected by CTEV are indicated in black.

Children affected by clubfoot and their parents were recruited through the United Kingdom support group for children with lower limb deformities, STEPS, and through the Dutch support group VOK (Fig 1). All families registered with STEPS and VOK as having a child affected by clubfoot were invited to take part in the study. Parents were contacted by mail and were asked whether they, and their affected child, would participate. Recruitment took place during 2001–2002. Fifty two percent of eligible families took part. Participants provided a buccal DNA sample, collected by mouthwash, or a cheek smear using a Cytocell brush (Medical Packaging Co., USA). Parents, and children who were old enough, provided their own sample. Samples from young children were collected by their parents using a cytocell brush. Mothers completed a questionnaire on socioeconomic factors, ethnicity, the pregnancy and birth of the index child, the nature of the child's clubfoot (laterality, etc.), the child's other medical conditions (to enable assessment of syndromic status), family history of clubfoot, maternal reproductive history, and maternal use of supplements and consumption of alcohol during the index pregnancy. CTEV phenotype was confirmed by asking parents to indicate which one of a series of four photographs of foot deformities most closely resembled their child's foot at birth, and by asking them to select one of four medical terms from a list, which included metatarsus varus, talus verticalis, ectrodactyly and oligodactyly, as well as congenital talipes equinovarus. Where a family history of CTEV was declared on the questionnaire, a study nurse (English or Dutch as appropriate) contacted the family by telephone. She sought further details of the phenotype to exclude non-CTEV foot deformity and to exclude syndromic CTEV in the proband. She then constructed a pedigree of at least three generations, explained our procedure for obtaining consent for the study, and arranged further mouthbrush DNA samples from siblings of the proband. If the proband's parent agreed to contact other affected family members and/or the parents as appropriate, they were sent a study pack to pass to their relative. This pack contained mouthbrush kits for the affected relatives, and other unaffected relatives such as parents. Affected relatives (or their parents in the case of children) were asked to complete a questionnaire, which included the same questions about phenotype (photographs and multiple choice medical terms) as administered to the proband. In this manner, we obtained DNA from at least two affected family members and the proband's parents from 91 families. These families comprised 91 affected probands, 22 affected, and 5 unaffected siblings of probands, 18 affected and 156 unaffected parents of probands, and 80 affected and 104 unaffected more distant relatives. In total, DNA samples from 476 individuals, 117 male and 94 female affected and 132 male and 133 female unaffected, were analysed in this study.

The study was approved by the Grampian Research Ethics Committee and the Medical Ethical Committee of the Academic Medical Center in Amsterdam.

### Genotyping methods

**Figure 2:**
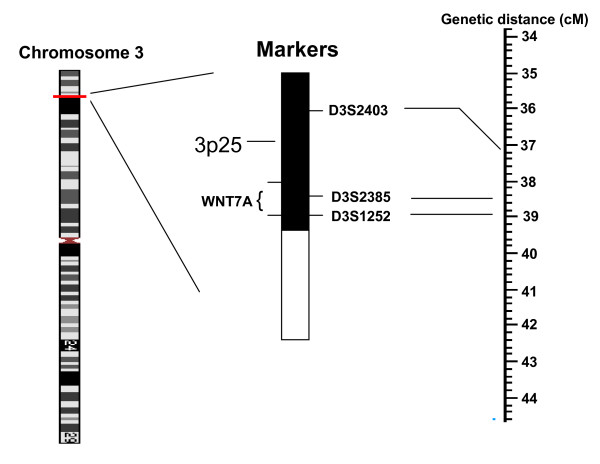
**Schematic representation of WNT7A chromosomal region**. Genetic map positions are from Marshfield [27].

DNA was extracted from the cheek smears and mouthwashes by using Instagene matrix (Bio-Rad, Hercules, California) and sodium hydroxide, respectively. Three short tandem repeat markers were selected for DNA amplification. D3S2403 and D3S1252 are linked to the gene WNT7A. D3S2403 is 700 kb 5' to the start of the gene while D3S1252 located 20 kb from the 3' end of the gene. D3S2385 is within an intron (Fig 2). The primers were fluorescence labelled (Sigma). Primer sequences are in Table 1. Amplification products were identified and quantified by use of capillary electrophoresis on an ABI 3100 sequencer and by use of GeneScan analysis software (version 3.7.1, ABI Biosystems).

**Table 1:** Details of STS markers linked to the WNT7A gene used in this study

Marker	Primer sequence	Gen Map* (cM)	Physical location (bp)**
D3S2403	F: ACAGATTGAGACCATGTGTCA R: CACACTCAAAATACATGAAGGC	37.20	13,147,397–13,147,709
D3S2385	F: GCTGTATTCGGGAGCATCTA R: CCACCATGAAAGAATGGCTA	38.54	13,853,945–13,854,287
WNT 7A			13,835,083–13,896,619
D3S1252	F: TGTGGCCACTGAACTCTCTG R: TCCAAGTGTTGAGAGCATGC	38.83	13,916,603–13,916,877

* Genetic map positions from Marshfield [27].

** Physical locations obtained through UCSC genome browser.

### Linkage analysis

Genotypes were analysed for linkage using the package GENEHUNTER [16]. Parametric and non-parametric models were used, and inter-marker genetic distances were estimated from physical map distances (1 cM/Mb). The parametric models were autosomal recessive and autosomal dominant with partial penetrance. The non-parametric model (NPL, non-parametric linkage) implemented by GENEHUNTER is described in Kruglyak *et al *[16], and is a development of previous affected-pedigree-member methods, allowing efficient use of multipoint data.

The marker allele frequencies were estimated from one randomly selected individual per pedigree. The frequencies listed in order of increasing allele size were as follows (frequencies set to 0.0001 are for alleles not observed in our sample, since a frequency of 0 is not allowed by GENEHUNTER):

D3S2403: 0.0001, 0.0001, 0.0173, 0.0878, 0.0216, 0.0001, 0.0043, 0.0288, 0.1685, 0.0894, 0.5763, 0.0057;

D3S2385: 0.0155, 0.2795, 0.472, 0.1988, 0.0342;

D3S1252: 0.69, 0.0001, 0.3099.

For the heterogeneity analysis, we used the facility of GENEHUNTER to optimise the value of alpha (fraction of linked families) to maximise the LOD score.

## Results

Genotyping of the three markers was performed in 476 individuals from 91 families, which included 211 affected cases. In the linkage analysis, we used both a simple recessive model of inheritance (genotype penetrances 0/0/1) and an incomplete penetrance dominant model (penetrances 0/0.33/0.33) since both models have been suggested as plausible by previous studies [3,17]. The data were also analysed using a non-parametric model. In this case, the information content of the analysis (calculated by GENEHUNTER) was between 0.27 and 0.29. In no case was any significant evidence for linkage observed. In fact, LOD scores for the parametric models remained strongly negative in the regions between the markers, and in the 0.5 cM intervals outside the marker map (Table 2). No significant lod scores were obtained when the data were analysed allowing for heterogeneity (results not shown). This result shows that the WNT7A gene is not a major cause of familial CTEV in this collection of families.

**Table 2:** Results of linkage analysis

Position (cM)*	Recessive Model LOD	Incomplete penetrance LOD**	Non-parametric score	Non-parametric p-value
-0.5	-42.225244	-33.909119	0.50113	0.287155
-0.4	-44.768435	-35.992476	0.50248	0.286652
-0.3	-47.971668	-38.616184	0.50384	0.286191
-0.2	-52.351361	-42.183565	0.5052	0.285689
-0.1	-59.533471	-47.885138	0.50656	0.285229
0	- infinity	-72.191026	0.50793	0.284728
0.14	-69.148113	-48.627625	0.57235	0.262384
0.28	-65.288751	-46.023141	0.63682	0.241056
0.42	-64.988176	-45.483255	0.70133	0.220786
0.56	-67.984864	-46.804102	0.76591	0.201567
0.7	- infinity	-58.961596	0.83053	0.183456
0.71	-94.432148	-52.757392	0.8011	0.191547
0.72	-91.779782	-51.295166	0.77166	0.199903
0.74	-91.558237	-50.687057	0.74221	0.208489
0.75	-93.691707	-50.70706	0.71276	0.217304
0.76	- infinity	-54.960933	0.68331	0.226347
0.86	-53.997125	-38.450649	0.68135	0.226971
0.96	-47.61052	-34.138501	0.6794	0.227558
1.06	-43.628903	-31.376063	0.67746	0.228183
1.16	-40.69302	-29.317393	0.67553	0.228773
1.26	-38.353692	-27.6683	0.6736	0.229363

* Positions in cM relative to D3S2403; D3S2385 and D3S1252 are at 0.7 and 0.76 cM respectively.

** Dominant model with penetrances of 0.33 for heterozygotes and homozygotes of disease allele.

## Discussion

WNT7A encodes a secreted protein that stimulates LMX-1 to confer dorsal patterning in the developing limb ectoderm [18]. The linkage findings of Dietz *et al*. [15] suggested WNT7A as a highly plausible candidate gene for CTEV. They used seven markers around WNT7A on chromosome 3 in a linkage study of a single large family. Marker D3S3608, about 0.16 Mb away from WNT7A, gave the highest LOD score of 2.18. We used one marker intragenic to WNT7A, one downstream, and one D3S2403 upstream of D3S3608 to ensure that the region surrounding D3S3608 was excluded. This marker also allowed us to exclude linkage to a gene upstream of WNT7A expressed in skeleton, FIBULIN 2. We found no evidence for linkage to any of these markers in this large study, and thus we have shown that variation in either WNT7A or FIBULIN 2 is very unlikely to be significant causes of familial CTEV.

Samples from the 91 families studied represented 168 affected, and 92 unaffected meioses. It is very unlikely that our ability to detect linkage was comprised by inadequate power, as if inheritance were autosomal dominant with perfectly informative markers, these meioses, could yield a lod of 50 or so at theta = 0. Analyses were performed using both plausible models of inheritance. Heterogeneity analysis gave no evidence that the linkage might be present in a sub-set of families. Such a linkage study cannot exclude genetic variation in WNT7A as a low penetrance risk factor for CTEV, nor can it exclude linkage in rare families or in populations other than those European populations studied. However, this candidate gene was proposed on the basis of such linkage analysis, and this study does exclude it as a major contributor to familial congenital talipes equinovarus.

To date, a small number of genes have been implicated in a small proportion of CTEV families. A mutation (R279W) in the diastrophic dysplasia sulphate transporter gene (DTDST) was reported as the aetiology of CTEV in two sets of siblings of western French ancestry [19], but this finding was not confirmed in a large series studied by transmission disequilibrium test for linkage and association [20]. A single missense mutation was once identified (M319K 956 T > A) in the homeodomain recognition helix of the HoxD 10 gene that segregated with disease in one large British family [21]. But after sequencing the HoxD 10 coding and 5' and 3' untranslated regions in 190 patients and linkage analysis in one large family, this gene was suggested not responsible for idiopathic clubfoot [22]. Heck *et al*. [23] identified a variant allele in the CASP10 gene that displays evidence of linkage and association with simplex CTEV cases. They further genotyped 40 SNPs spanning seven apoptotic genes including CASP10 in 210 simplex trios and 139 multiplex families confirming that the variation in these genes may play a role in development of clubfoot [24]. We recently found that polymorphism of the methylenetetrahydrofolate reductase gene (MTHFR) was associated with clubfoot [25]. Also recently N-acetylation genes, *NAT1 *and *NAT2 *were reportedly associated with CTEV [26]. Despite rapid recent progress in the molecular basis of CTEV, a "major gene" as suggested by the segregation analyses remains elusive. The best way forward for CTEV research may be collaborative studies to perform high density genome-wide scans for linkage and association.

## Conclusion

Our evidence suggests that the WNT7A gene is unlikely to be a major contributor to the aetiology of familial CTEV.

## Competing interests

The authors declare that they have no competing interests.

## Authors' contributions

ZM and LS designed the ECCE study and led its conduct. ZM had the idea to study Wnt-7a. GL, JI and SS selected genetic markers for analysis, designed and performed the molecular assays. ZM, AC, RH and LS designed and implemented the sample collection. DS performed and interpreted the linkage analysis. GL and ZM drafted the paper with input from all authors.

## Pre-publication history

The pre-publication history for this paper can be accessed here:


